# Prevalence of High Jugular Bulb across Different Stages of Adulthood in A Chinese Population

**DOI:** 10.14336/AD.2020.0215

**Published:** 2020-07-23

**Authors:** Jingjing Wang, Yanmei Feng, Hui Wang, Chunyan Li, Yaqin Wu, Haibo Shi, Shankai Yin, Zhengnong Chen

**Affiliations:** ^1^Department of Otolaryngology-Head and Neck Surgery, Shanghai Jiao Tong University Affiliated Sixth People's Hospital, Shanghai 200233, China; ^2^Otolaryngology Institute of Shanghai Jiao Tong University, Shanghai 200233, China; ^3^Shanghai Key Laboratory of Sleep Disordered Breathing, Shanghai 200233, China

**Keywords:** high jugular bulb, jugular bulb, age, prevalence, Chinese population

## Abstract

Pulsatile tinnitus, ear fullness, vertigo, hearing disorders, and vestibular dysfunction have been found to be related to high jugular bulb. Anatomical variation in this region also affects surgical planning and approaches. Therefore, knowledge on the detailed anatomy of the high jugular bulb is critical for middle ear and lateral skull base surgery. Prevalence of high jugular bulb is uncertain as data are usually derived from temporal bone specimens and patient reports from hospitals. Therefore, a community-based epidemiological study is necessary to understand the significance of high jugular bulb anatomy. Here, we report a cross-sectional study to characterize the prevalence of high jugular bulb and jugular bulb size using a 3.0 T magnetic resonance imaging. Furthermore, we studied the relationship between the prevalence of high jugular bulb and age-related changes. We enrolled 4539 permanent residents (9078 ears) from two communities in the Shanghai region who underwent magnetic resonance imaging between 2007 and 2011. We divided participants into four subgroups according to age: 35-44 (early middle age), 45-54 (middle age), 55-64 (late middle age), and 65-75 (late adulthood) years. We found that the overall prevalence of high jugular bulb was 14.5% in a Chinese population. There was a higher prevalence of high jugular bulb on the right side and especially in women (both *p* < 0.001). The occurrence of high jugular bulb was higher in the early middle age group and gradually decreased with age, but was still present in the late adulthood group (*p* = 0.039). These findings provide useful information on the prevalence of high jugular bulb in a Chinese population and the distribution in age groups, suggesting that high jugular bulb should be considered, even in those without ear disorders. This work serves as a foundation for further research on the relationship between jugular bulb changes and disease symptoms.

Jugular bulb (JB) is the jugular vein blood vessels in a corner of extracranial, shaped like a ball, with its wall thin and vulnerable. JBs are surrounded by the bony jugular fossa, which lies below the temporal bone [1]. The dome of the JB extends more superiorly in the petrous temporal bone. The anatomy of JBs vary; JBs that lie higher than the inferior aspect of the internal auditory canal are known as high jugular bulbs (HJBs) [2-6]. JB abnormalities include HJB, JB dehiscence, and JB diverticulum [2, 7]. HJB is the most common and clinically important anatomical variation of the temporal bone, and often impedes diagnoses, treatment planning, and surgical interventions [2, 6-12].

HJB is thought to underlie the presentation of certain clinical symptoms, including pulsatile tinnitus, ear fullness, vertigo, hearing disorders, and vestibular dysfunction, among others [6, 8, 11, 13-17]. Conductive deafness occurs when the HJB obstructs the round window, protrudes against the pars tensa, or makes occasional contact with the ossicular chain [8, 18, 19]. Sensorineural hearing disorders and tinnitus may further result from close contact between the HJB and the vestibulocochlear nerve in the internal auditory canal, as well as JB protrusion into the inner ear via the round window [8, 18-20]. Moreover, an HJB may cause vertigo and other vestibular dysfunctions by preventing the vestibular aqueduct from reabsorbing endolymph, which subsequently causes Meniere’s disease [8, 21]. Given these relationships to clinical disease, it is important to obtain more knowledge of the anatomy of HJBs.

Previous studies have reported that JB development continues for an extended period of time postnatally [22-24]. This postnatal development may sometimes result in increased HJB prevalence and JB enlargement. JB development continues until the age of 40 years, after which the JB size becomes relatively stable [22-24]. Previous studies that report the prevalence of HJB are usually based on studies of temporal bone specimens or patient reports from hospitals. As a result, the prevalence of HJB may be overestimated due to the selection of a patient sample with related clinical symptoms [1, 4-6, 22, 23]. These studies are also limited either due to sampling bias, a small sample size, or both [1, 4-6, 22, 23]. Currently, there is no strong evidence to suggest that the JB remains stable after 40. In addition, there are no reports of the occurrence of HJB or JB size in a healthy population above the age of 40. Although one prior histopathologic study reported the frequency of JB abnormalities by decade of life, studies which exclusively focus on the age-related prevalence of HJB are lacking [23]. Given these gaps in the literature, we conducted a community-based epidemiological study to examine the prevalence of HJB in a healthy population between the ages of 35 and 75 years using a 3.0 T magnetic resonance imaging (MRI) scanner. We further determined JB size as well as other factors in the various age groups. Lastly, we emphasised the significance of HJB anatomy for middle ear and lateral skull base surgery. Otologists should be aware of variations in HJB anatomy in particular populations.

## MATERIALS AND METHODS

### Ethical considerations

The present study protocol was approved by the Institutional Review Board of the Sixth People’s Hospital Affiliated to Shanghai Jiao Tong University, Shanghai, China. All study methods were performed in accordance with the relevant guidelines and regulations. All participants provided informed consent prior to study enrollment.

**Table 1:** Study participant characteristics.

Characteristics	Total (N=4539)	Normal jugular bulb (normal JB, n=3883)	High jugular bulb (HJB, n=656)	P value^*^
Mean age (SD), y	55.5 (10.1)	55.6 (10.1)	54.6 (10.1)	0.027^*^
Female/Male, n/n	2395/2144	2005/1878	390/266	<0.001^*^
Mean body mass index (SD), kg/m^2^	23.6 (3.0)	23.7 (3.0)	23.5 (3.1)	0.049^*^
Smoking, n (%)^+^	981 (21.6%)	859 (22.1%)	122 (18.6%)	0.043^*^
Alcohol use, n (%)^#^	569 (12.5%)	492 (12.7%)	77 (11.7%)	0.505
Hypertension, n (%)	1147 (25.3%)	999 (25.7%)	148 (22.6%)	0.084
Diabetes, n (%)	329 (7.2%)	278 (7.2%)	51 (7.8%)	0.574
Hyperlipidemia, n (%)	357 (7.9%)	311 (8.0%)	46 (7.0%)	0.380
Stroke, n (%)	134 (3.0%)	116 (3.0%)	18 (2.7%)	0.733
Coronary heart disease, n (%)	96 (2.1%)	87 (2.2%)	9 (1.4%)	0.153
Myocardial infarction, n (%)	16 (0.4%)	15 (0.4%)	1 (0.2%)	0.563
Arrhythmia, n (%)	84 (1.9%)	75 (1.9%)	9 (1.4%)	0.325

* *p* < 0.05, comparisons between normal JB and HJB.

+ Smoked >100 cigarettes in a lifetime.

# Consumption of > 30g of alcohol per week for longer than 1 year. SD = standard deviation.

### Study design

Permanent residents of two communities in the Shanghai region participated in the study. Participants with cochlear implants, otitis media, implanted pacemakers, intraocular metal foreign bodies, inner ear implants, metal prostheses, or ferromagnetic foreign bodies were excluded. Exclusion criteria also included participants with early pregnancy or claustrophobia, as well as those who did not adhere to the study protocol [25].

The general information of participants was obtained, including height, body weight, smoking status (defined as smoking >100 cigarettes in a lifetime), and alcohol consumption (defined as the consumption of >30 grams of alcohol per week for longer than 1 year), as well as the presence or history of hypertension, diabetes, hyperlipidemia, stroke, coronary heart disease, myocardial infarction, and arrhythmia [25]. Furthermore, we calculated each participant’s body mass index (BMI). Based on the recommendations issued by the Working Group on Obesity in China, we defined obesity as a BMI great than 28 kg/m^2^.

All enrolled participants underwent a head MRI scan at some point between 2007 and 2011 at the Department of Radiology, the Sixth People's Hospital Affiliated to Shanghai Jiao Tong University, China. MRIs were acquired using an Achieva 3.0 T MRI system (Philips Healthcare, Amsterdam, The Netherlands). We detected HJBs with high resolution MRI instead of CT because MRI has no x-ray. Participants were divided into four subgroups according to their age, as follows: 35-44 (early middle age), 45-54 (middle age), 55-64 (late middle age), and 65-75 (late adulthood) years. All axial and coronal MR images were measured and analysed by one senior radiologist and assessed by one experienced otologist [26].

**Figure 1.**
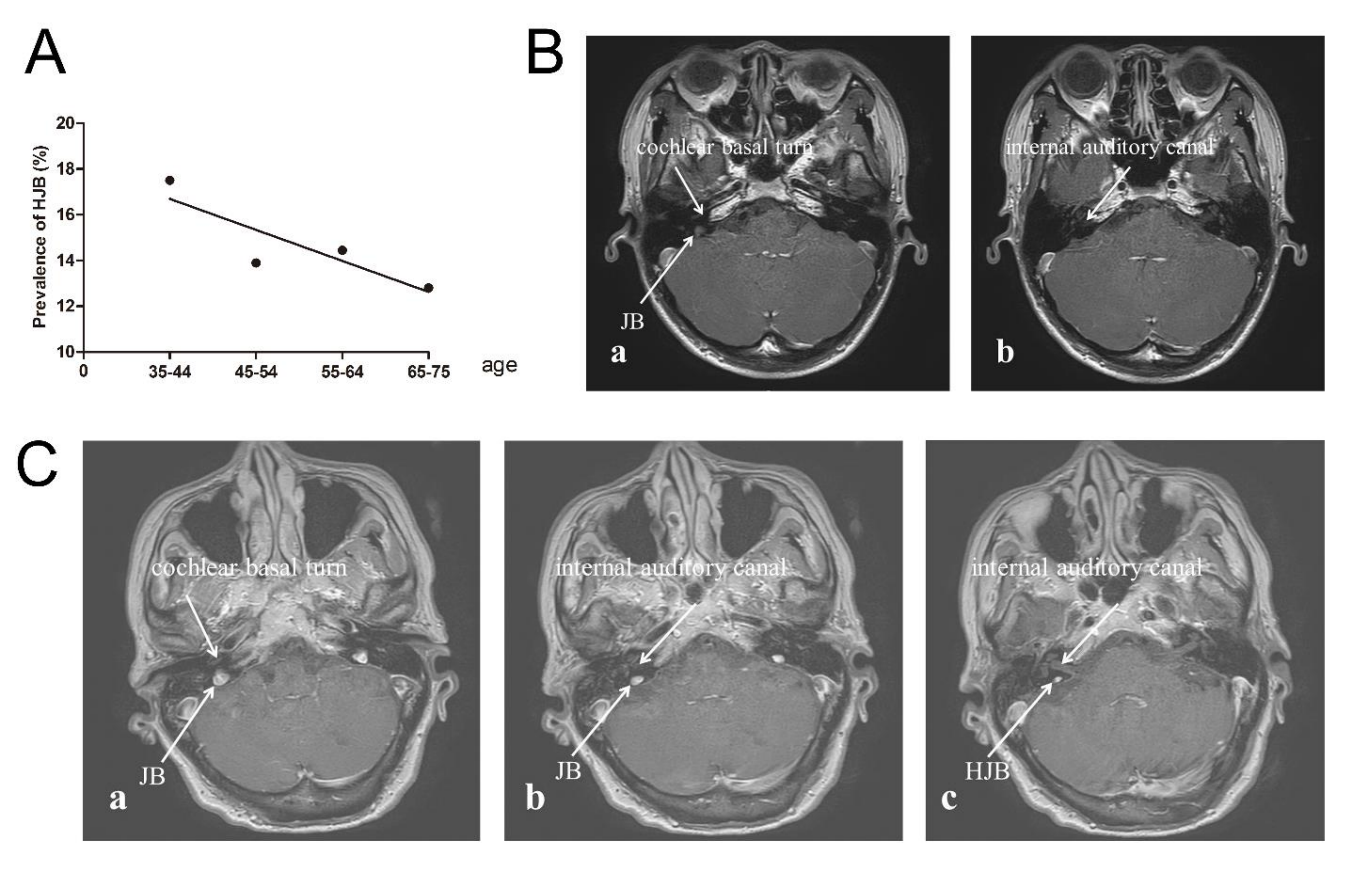
**Age-related HJB prevalence and MR images of different JB types**. **(A)** The prevalence of HJB (%) across age groups. **(B)** Axial MR images showing dynamic changes in the normal JB. (a) A JB reaching the cochlea basal turn; (b) the JB was not seen at the level of the internal auditory canal. **(C)** Axial MR images showing dynamic changes in the HJB. (a) A JB reaching the cochlea basal turn; (b) a JB rising above the cochlear basal turn and inferior to the internal auditory canal; (c) the HJB extending superiorly to the internal auditory canal. HJB = high jugular bulb; MR = magnetic resonance; JB = jugular bulb.

As reported previously, we defined HJB as a JB that lies higher than the inferior aspect of the internal auditory canal on MR images (Fig. 1) [2-4]. We obtained JB measurements in the axial plane from its greatest cross-sectional area [13, 22, 23]. On axial images, bilateral JBs were measured in the anteroposterior and mediolateral planes in millimeters [13, 22, 23]. Given the elliptical configuration of JBs, we calculated the JB area using the following formula: Area = π * A * B, where A and B are one-half of the ellipse’s major and minor axes, respectively; A = (1/2)* (anteroposterior measurement) and B = (1/2)* (mediolateral measurement) [13, 22, 23]. We made all numerical measurements on axial images on a computer monitor via manual placement of a measurement cursor. All measurements are in millimetres.

### Statistical analyses

We used SPSS statistical software (version 20 for Windows; IBM Corp., Armonk, NY) to analyse all data. P-values < 0.05 were considered statistically significant for all analyses.

## RESULTS

In total, 4539 participants (9078 ears; 2144 men and 2395 women) were enrolled in the study. The prevalence of HJB in each age group was as follows: 17.6% (135/769, 35-44 years, early middle age), 13.9% (215/1544, 45-54 years, middle age), 14.5% (180/1245, 55-64 years, late middle age), and 12.8% (126/981, 65-75 years, late adulthood). The prevalence of HJB decreased with age (trend test, *p* = 0.039; Fig. 1). Furthermore, the prevalence of HJB was greater in women than in men (*p* < 0.001, Table 1). We observed bilateral HJB in 82 (1.8%) participants; 20 (24.4%) men and 62 (75.6%) women. Age stratification into two groups revealed that the prevalence of HJB in participants less than 60 years of age was 14.8% (439/2964), and 13.8% (217/1575) in participants ≥ 60 years of age.

Table 2 shows JB sizes in relation to age. The area of the left JB in each age group was as follows: 47.4 (25.7) mm^2^ [35-44 years], 46.2 (25.4) mm^2^ [45-54 years], 44.5 (25.9) mm^2^ [55-64 years], and 42.5 (25.7) mm^2^ [65-75 years]. There was a decrease in left JB size with increasing age (p < 0.001). The areas of the right JB were 58.6 (30.9) mm^2^, 57.3 (30.8) mm^2^, 59.6 (32.5) mm^2^, and 56.3 (30.5) mm^2^ in the age groups of 35-44, 45-54, 55-64, and 65-75 years, respectively. There was an unclear trend in the relationship between the area of the right JB and age (p = 0.312). Moreover, the average area of the right JB (58.0 mm^2^) was larger than the average area of the left JB (45.1 mm^2^, p < 0.001; Table 2).

**Table 2:** Age- and sex-specific area of the JB [normal jugular bulb (normal JB) + high jugular bulb (HJB)].

Age (yrs)	Men				Women				All			
	Participants (N)	Area (mm^2^)		P_1_	Participants (N)	Area (mm^2^)		P_2_	Participants (N)	Area (mm^2^)		P_3_
		Left	Right			Left	Right			Left	Right	
35-44	384	47.6 (23.2)	59.7 (30.0)	<0.001	385	47.3 (27.9)	57.6 (31.8)	<0.001	769	47.4(25.7)	58.6(30.9)	<0.001
45-54	686	47.8 (25.6)	59.4 (31.3)	<0.001	858	44.9 (25.1)	55.7 (30.3)	<0.001	1544	46.2(25.4)	57.3(30.8)	<0.001
55-64	577	45.9 (26.7)	62.3 (33.6)	<0.001	668	43.2 (25.1)	57.3 (31.3)	<0.001	1245	44.5(25.9)	59.6(32.5)	<0.001
65-75	497	42.4 (25.4)	56.4 (29.5)	<0.001	484	42.7 (26.1)	56.2 (31.4)	<0.001	981	42.5(25.7)	56.3(30.5)	<0.001
Total	2144	46.0 (25.5)	59.5 (31.3)	<0.001	2395	44.3 (25.8)	56.6 (31.1)	<0.001	4539	45.1(25.7)	58.0(31.2)	<0.001

P_1_: comparison of lateralised areas of JB in men across age groups. P_2_: comparison of lateralised areas of JB in women across age groups. P_3_: comparison of lateralised areas of JB across all age groups.

We detected right-sided HJB in 455 (10.0%) participants, which included 188 (41.3%) men and 267 (58.7%) women. We detected left-sided HJB in 283 (6.2%) participants, including 98 (34.6%) men and 185 (65.4%) women. Overall, the prevalence of HJB was higher in the right ear and in women (both *p* < 0.001, Table 1). Additionally, for all ears, the average HJB area (65.2 mm^2^) was found to be larger than the average of the normal JB area (49.2 mm^2^, *p* < 0.001; Table 3).

## DISCUSSION

In the present study, we found that the prevalence of HJB was 14.5% (656/4539) in a large, healthy Chinese population. There was a higher prevalence of HJB on the right side and in women (both *p* < 0.001). The occurrence of HJB was highest in the 35- to 45-year-old age group, and gradually decreased with age, but was still prevalent in the over 65-year-old age group (12.8%; *p* = 0.039). These findings provide useful information regarding the prevalence of HJB in a Chinese population as well as the distribution between age groups; overall, these findings indicate that the existence of HJB should be considered, even in those without ear disorders.

We found that the HJB area was significantly larger than the normal JB area, indicating that decreased HJB prevalence may result from smaller JB size. However, we found lateral differences in JB size with increasing age. Specifically, there was a significant decrease in the size of the left JB with increasing age, whereas the right JB, which had a higher HJB prevalence and was often larger than the left, did not decrease with increasing age.

**Table 3:** Age- and sex-specific area of different JB types [normal jugular bulb (normal JB) or high jugular bulb (HJB)].

Age (yrs)	Men					Women					All				
	Normal JB		HJB		P_1_	Normal JB		HJB		P_2_	Normal JB		HJB		P_3_
	Ears (n)	Area (mm^2^)	Ears (n)	Area (mm^2^)		Ears (n)	Area (mm^2^)	Ears (n)	Area (mm^2^)		Ears (n)	Area (mm^2^)	Ears (n)	Area (mm^2^)	
35-44	652	52.0 (25.4)	116	62.6 (36.2)	0.013	616	49.8 (28.6)	154	62.9 (34.6)	<0.001	1268	51.0 (27.0)	270	62.8 (35.2)	<0.001
45-54	1210	52.0 (27.9)	162	65.8 (34.7)	<0.001	1448	47.6 (26.5)	268	64.8 (33.1)	<0.001	2658	49.6 (27.2)	430	65.2 (33.7)	<0.001
55-64	1018	52.2 (29.1)	136	68.8 (42.4)	<0.001	1112	47.2 (27.0)	224	65.1 (35.0)	<0.001	2130	49.6 (28.1)	360	66.5 (37.9)	<0.001
65-75	876	47.5 (27.0)	118	63.6 (34.0)	<0.001	834	46.5 (26.9)	134	68.0 (38.0)	<0.001	1710	47.0 (27.0)	252	65.9 (36.2)	<0.001
Total	3756	51.0 (27.7)	532	65.4 (36.9)	<0.001	4010	47.6 (27.1)	780	65.1 (34.8)	<0.001	7766	49.2 (27.4)	1312	65.2 (35.7)	<0.001

P_1_: comparison of normal JB and HJB areas in men across age groups. P_2_: comparison of normal JB and HJB areas in women across age groups. P_3_: comparison of normal JB and HJB areas in all participants across all age groups.

There is huge variation and uncertainty in the reported prevalence rates of HJB, with estimates ranging from 2.8% to 65% [5, 8, 10, 19, 21, 27-30]. We believe that the variation is due to a lack of consistency in the definition of HJB across studies, as well as variations in sample size, among other factors. Moreover, sex and age distribution differences across study populations, as well as differences in research techniques and study designs, could influence findings [25].

Age has been found to contribute to changes in the prevalence of HJB and in JB size. For instance, previous studies indicate that JBs are not present at birth and first appear at two years of age, increase in size during the first 40 years of life, and then stabilise [22-24]. The frequency of HJB was found to be lowest in the first decade of life (1.7%) and then increases during the subsequent four decades of life, remaining at approximately 8-11% after the fifth decade of life (41-50 years) [23]. Although previous studies suggest that JB size stabilises after 40 years of age, we found continued change after the age of 35. Specifically, we found that the prevalence of HJB decreased from 17.6% at the ages of 35-44 years to 12.8% at the ages of 65-75 years. Similarly, the size of the left JB decreased with age (47.4 mm^2^ to 42.5 mm^2^). A previous study further demonstrated an association between bony resorption and HJB [4]. Based on these findings, we speculate that bony resorption, which decreases with the aging process, subsequently leads to a decreased prevalence of HJB. Additionally, mastoid pneumatisation and temporal bone development might play an important role [23].

To the best of our knowledge, no previous studies have assessed the independent factors that affect the occurrence of HJB. Here, we used a logistic regression analysis adjusted for age, sex, BMI, smoking, alcohol consumption, hypertension, diabetes, hyperlipidemia, stroke, coronary heart disease, myocardial infarction, and arrhythmia, and found that age and sex were independent predictive factors of HJB. Specifically, we found that younger age and female sex were independently associated with an increased HJB prevalence. Differences in the prevalence of HJB could therefore be associated with the aging process, given that we excluded sex. Although a large proportion of participants had hypertension and unhealthy living habits (e.g., smoking or alcohol use), the association between these risk factors and the prevalence of HJB was not statistically significant. Future longitudinal studies should further explore this association.

As stated above, the present study further revealed that the prevalence of HJB was higher on the right side and in women. Asymmetry and right-sided dominance of the jugular vein ball under normal conditions has been reported previously, which is consistent with our own findings [3, 6, 12, 13, 16, 19, 21, 26, 30]. Increased length of the left brachiocephalic vein could further increase the intravenous pulsation energy generated by the heart and facilitate the formation of a larger right-sided JB [22, 29]. Alternatively, this right-sided dominance could be linked to asynchronism of the embryonic venous sinuses, and thus, to asymmetric flow during the early stages of cardiac venous pulsation [22]. Furthermore, and in agreement with the present study, previous studies have also reported a significantly higher prevalence of HJB in women [6, 11, 31]. These studies suggest that hormone levels could contribute to this increased prevalence. The mechanisms underlying the age-related changes in JB size remain largely unknown; however, changes in hormone levels may be involved.

One of the limitations of HJB assessments was the difficulty in executing the translabyrinthine approach. Sufficient exposure of the cochlear aqueduct is extremely important during translabyrinthine microsurgery since it is necessary for opening the cerebellomedullary cistern, achieving cerebrospinal fluid release, and recognising the posterior cranial nerves [29]. The presence of HJB narrows the surgical field and therefore limits exposure of the cochlear aqueduct. Furthermore, when the JB is above the internal auditory canal, it is extremely difficult to fully expose the surgical field through downward pressure on the JB with bipolar forceps, a detacher, and bone wax. Inadvertent tearing of the JB is dangerous and may cause life-threatening haemorrhages, as well as increases the risk for air embolism [8, 29]. This is a relative and sometimes absolute contraindication for the translabyrinthine approach to vestibular schwannoma resections. The translabyrinthine approach may therefore be inappropriate in such cases. Given this, HJBs affect the choice of surgical approach [8, 29].

Here, we found that the area of HJBs was larger than that of normal JBs, regardless of age or sex. This result is consistent with prior work and suggests that there is some volumetric expansion during the transition from normal JBs to HJBs; however, the etiology of this expansion remains unknown [23]. HJBs not only have a higher position, but also occupy a larger area. Moreover, the higher the JB position, the thinner is its wall [4]. Significant and life-threatening haemorrhages may occur upon HJB damage; therefore, patients with HJBs should receive special attention from otologists.

Despite its contributions to the field, the present study was limited because it only included adults between the ages of 35 and 75, thus limiting the generalisability of the study findings to additional age groups. Compared with previous studies, however, the present study enrolled a larger number of participants (N = 4539). While previous studies assessed temporal bone specimens or patients enrolled from medical centres or hospitals, we enrolled healthy participants from a community-based population, thus reducing the potential for sample bias [14, 22, 23]. Given that HJB may underlie the presentation of certain clinical symptoms and affect surgical approach, and that prior studies have reported varied HJB prevalence values, the large community-based epidemiological approach used here was an essential contribution to the field [5, 6, 8, 10, 11, 13-17, 19, 21, 27-30]. In addition, we also further utilised objective measures, rather than rough estimates or relative measurements. Given these advantages, our study findings are valuable and could contribute to further research on the relationship between changes in JB and disease symptomatology.

In conclusion, the present study provides useful evidence of changes in the JB up until the age of 75. Critically, we report a decreased prevalence of HJB and left-lateralised JB size with increasing age. This suggests that age should be considered when reporting the prevalence of HJB. Further studies should clarify the mechanisms underlying this phenomenon.
